# A revision of the genus *Kaszabister* Mazur (Histeridae, Histerinae, Exosternini)

**DOI:** 10.3897/zookeys.199.3245

**Published:** 2012-06-04

**Authors:** Nicolas Dégallier, Sławomir Mazur, Alexey K. Tishechkin, Michael S. Caterino

**Affiliations:** 1 120 rue de Charonne, 75011 Paris, France; 2 Katedra Ochrony Lasu i Ekologii, Warsaw University of Life Sciences, Nowoursynowska 159/34, 02-776 Warszawa, Poland; 3 Department of Invertebrate Zoology, Santa Barbara Museum of Natural History, 2559 Puesta del Sol, Santa Barbara, California 93105 USA

**Keywords:** Histeridae, Exosternini, *Kaszabister*, myrmecophily, *Solenopsis*, Neotropical Region

## Abstract

We revise the four species of *Kaszabister* Mazur, 1972, one of which, *Kaszabister barrigai* **sp. n.**, is described as new. The other species in the genus are *Kaszabister rubellus* (Erichson, 1834), *Kaszabister ferrugineus* (Kirsch, 1873) and *Kaszabister carinatus* (Lewis, 1888). The species are principally known from the subtropics of South America, with one in Central America. Lectotypes are designated for *Kaszabister rubellus* and *Kaszabister ferrugineus*, and a key is provided for all the species. Ants of the genus *Solenopsis* Westwood, mainly *Solenopsis invicta* Buren and *Solenopsis saevissima* (Smith), are documented as hosts of three of the four species.

## Introduction

The genus *Kaszabister* Mazur, 1972, was initially described for a single species, now known as *Kaszabister rubellus* (Erichson, 1834), and assigned to the myrmecophilous and termitophilus subfamily Haeteriinae. Later, two other species originally described in other genera (*Epierus ferrugineus* Kirsch, 1873, and *Phelister carinatus* Lewis, 1888) were moved here, and the entire genus was moved to Histerinae: Exosternini (Mazur 1997). This dynamic taxonomic history underscores the enigmatic nature of this genus, and its affinities within the tribe remain unclear. Similarly, little is known about the biology of these species, although it has become clear that they live as guests in the nests of fire ants of the genus *Solenopsis* Westwood. These ants are a major nuisance as a result of invasion into many tropical and subtropical climates (Hölldobler and Wilson 1990). It is therefore important to better understand the commensal predators that may play a role in controlling ant populations.

This paper represents the first installment of an ongoing revision of all the species of New World Exosternini. The fauna is large and complex, with the limits of most genera poorly understood. *Kaszabister* is among the more straightforward and clearly monophyletic groups, and preliminary analyses (Caterino et al. in prep) indicate that it lies outside any other named genus. Thus we are confident in retaining its status as a genus.

## Materials and methods

The morphological terminology used is that defined by Wenzel and Dybas (1941), supplemented by Helava et al. (1985), Ôhara (1994) and Lawrence et al. (2011). Following histerid conventions, total body length is measured from the anterior margin of the pronotum to the posterior margin of the elytra (to exclude preservation variability in head and pygidial extension), while width is taken at the widest point, generally near the elytral humeri. Type material of all valid species was examined by one or more of the authors. Photographic imaging was done using a Visionary Digital’s ‘Passport’ portable imaging system, which incorporates a Canon D7 with MP-E 65mm 1–5X macro zoom lens. Images were stacked using Helicon Focus software. SEM imaging was done on a Zeiss EVO 40 scope, with most specimens sputter coated with gold. Photographs of all type specimens are available through the Encyclopedia of Life (www.eol.org).

Specimens from the following institutions were utilized:

BMNH The Natural History Museum, London, UK

CHAT The Alexey Tishechkin Collection, Santa Barbara, USA

CHND The Nicolas Degallier Collection, Paris, France

CHPK The Piet Kanaar Collection, Leiden, The Netherlands

CHSM The Slawomir Mazur Collection, Warsaw, Poland

DBIA University of Brasilia, Distrito Federal, Brazil

EMEC The Essig Museum of Entomology, Berkeley, USA

FMNH The Field Museum, Chicago, USA

FSCA Florida State Collection of Arthropods, Gainesville, USA

HNHM Hungarian Natural History Museum, Budapest, Hungary

INBI Instituto Nacional de Biodiversidad, San Jose, Costa Rica

MACN Museo de Ciencias Naturales “Bernardino Rivadavia”, Buenos Aires, Argentina

MNHN Museum National d’Histoire Naturelle, Paris, France

NMNH National Museum of Natural History, Washington, USA

SMTD Staatlichen Museum für Tierkunde, Dresden, Germany

ZHMB Museum für Naturkunde, Berlin, Germany

## Taxonomy

### Genus Kaszabister

Mazur, 1972

http://species-id.net/wiki/Kaszabister

Kaszabister Mazur, 1972: 189.

#### Type species.

*Kaszabister mahunkai* Mazur, 1972 (now regarded as a junior synonym of *Kaszabister rubellus* (Erichson, 1834)), original designation.

#### Diagnosis.

**Figure 1.**
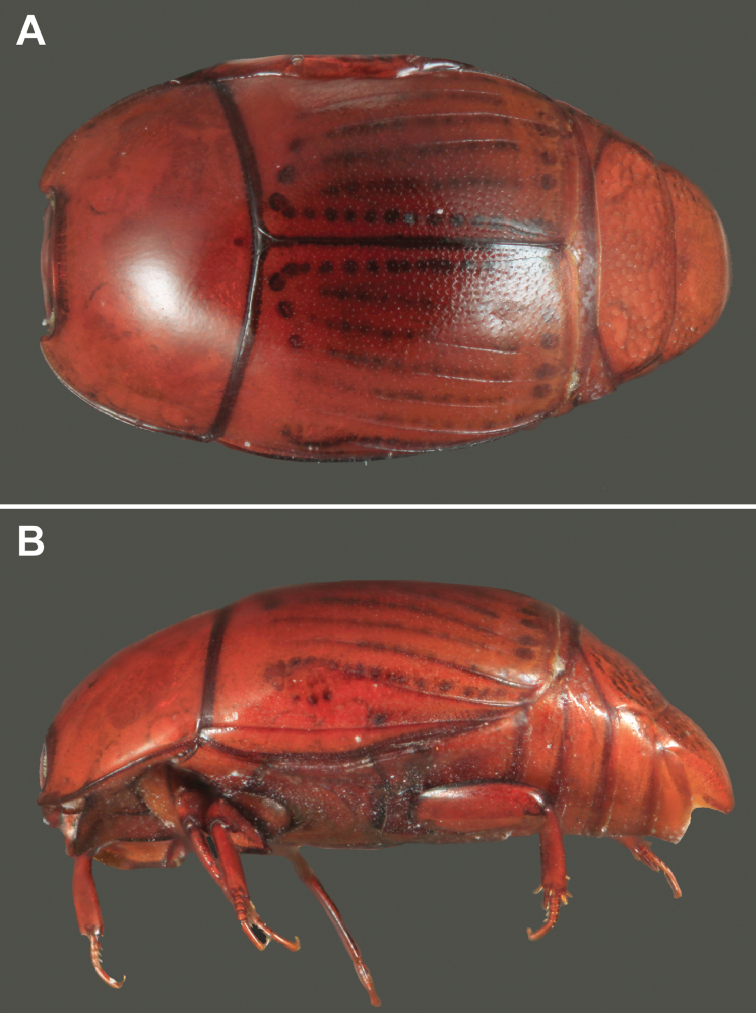
Habitus photos of *Kaszabister barrigai* sp. n. **A** Dorsal **B** Lateral.

**Figure 2.**
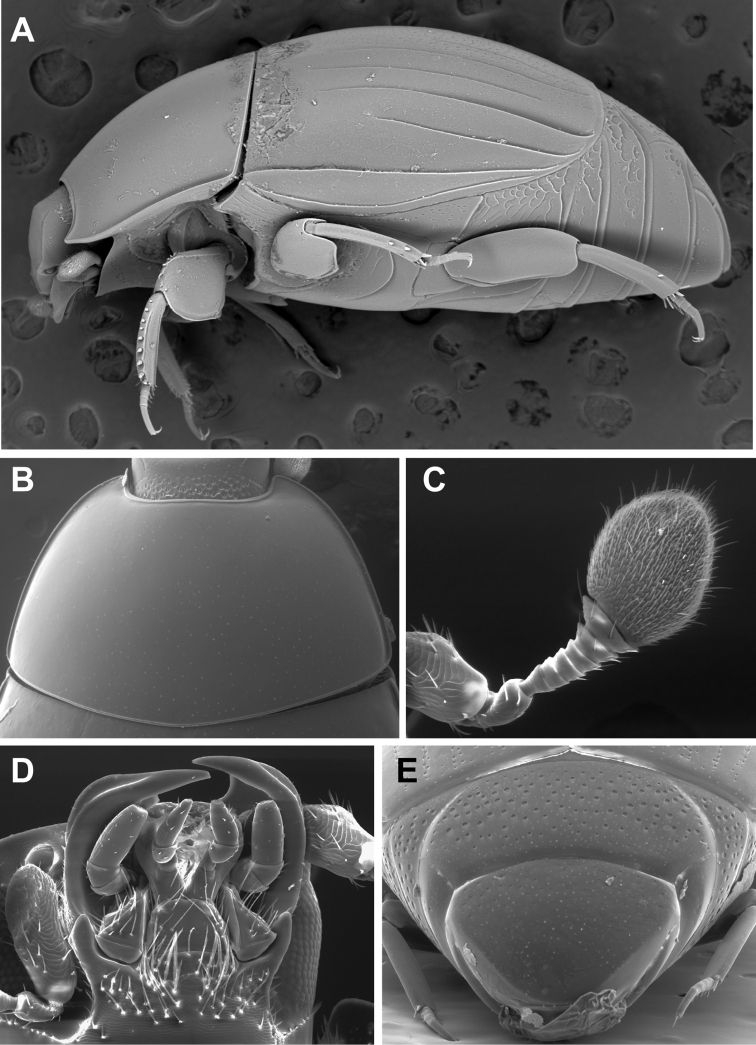
Generic characters of *Kaszabister*. **A** Lateral habitus of *Kaszabister barrigai* showing carinate and convergent dorsolateral elytral striae **B** Pronotum of *Kaszabister carinatus* **C** Antenna of *Kaszabister carinatus* **D** Mouthparts of *Kaszabister carinatus* **E** Propygidium and pygidium of *Kaszabister carinatus*.

**Figure 3.**
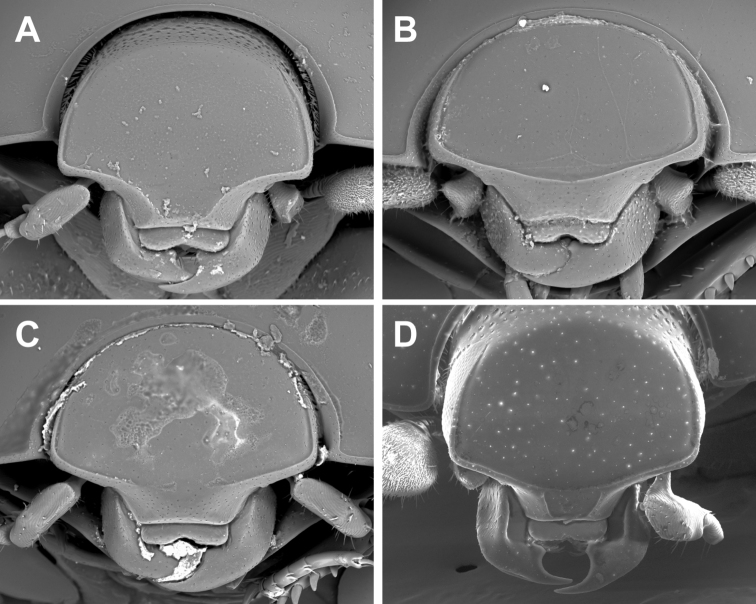
Anterior view of head. **A** *Kaszabister barrigai* **B** *Kaszabister ferrugineus* **C** *Kaszabister rubellus* **D** *Kaszabister carinatus*.

**Figure 4.**
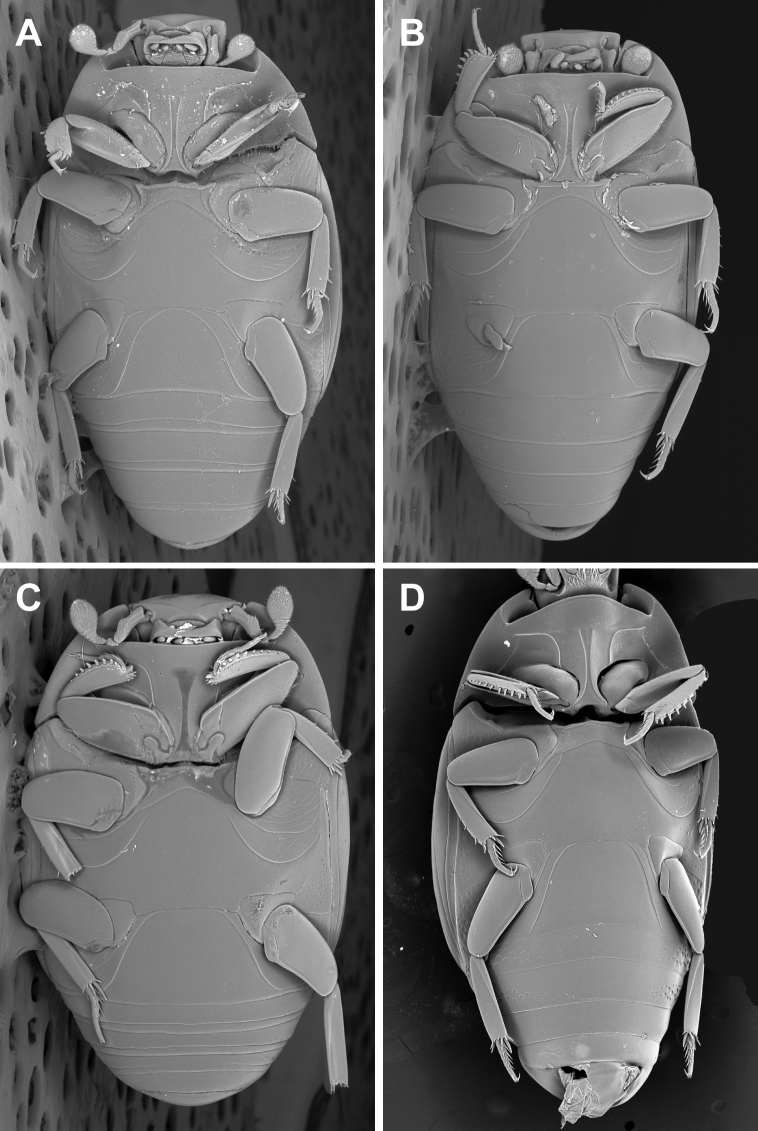
Ventral habitus. **A** *Kaszabister barrigai* **B** *Kaszabister ferrugineus* **C** *Kaszabister rubellus* **D** *Kaszabister carinatus*.

*Kaszabister* can be easily separated from other Neotropical Exosternini by its strongly carinate frontal stria (Fig. 3); epipleural, subhumeral, and dorsal elytral striae 1 apically carinate and convergent to posterolateral corner (Fig. 1B, 2A); and narrow, edentate meso- and metatibiae which bear only a few small spines (Figs 1B, 2A, 4). The narrowly depressed lateral pronotal margin is also rare in other genera (Figs 1A, 2B).

#### Description.

Body length 1.7–2.3mm, width 1.3–1.7mm, oval or oblong, more or less convex, reddish brown, glabrous. ***Head*:** Frons bordered by a prominent, moderately to strongly carinate frontal stria; antennae inserted under the rim of the frons in front of eyes; antennal scape slightly setose; antennal club oval, tomentose, lacking sutures or annuli, with small oval subapical sensoria on upper and lower surfaces; epistoma flat to convex, bordered by striae or carinae; labrum short, broad, rounded at sides and emarginate at middle; mandibles with strong furrows along lower outer margins and very weak subapical teeth on incisor edge; gena setose and weakly depressed; gular sutures impressed; submentum with numerous fringed setae, projecting slightly between maxillar bases; mentum about one-fourth broader than long, sides rounded, tapering apically, margin faintly emarginate; palpi relatively short, with truncate apices. ***Pronotum*:** pronotum widest at base, sides rounded, anterior angles acute; prescutellar impression absent; gland openings annulate, situated about one-third from anterior margin, behind inner edge of eye on each side; with 3 pores along each side; marginal stria complete, continuous with anterior marginal stria; lateral stria absent. ***Elytra*:** Dorsal striae of elytra simple or carinate, variously abbreviated; dorsal stria 1, subhumeral striae, and epipleural stria carinate and convergent apically. ***Prosternum*:** Antennal cavities of the prosternum visible in ventral view, located in the anterior angles of pronotum; prosternal lobe short, broad, reaching hypomeron laterally, with marginal stria at least medially; base of prosternal keel weakly emarginate; with complete carinate striae diverging anterad and posterad, not joined. ***Mesoventrite*:** Disk flat, weakly projecting at middle, with complete marginal stria; mesometaventral stria present, angulate forward onto mesoventral disk. ***Metaventrite*:** Metaventral disk with postcoxal and lateral striae, both extending laterad toward metepisternum. ***Abdomen*:** Propygidium short, moderately convex, with annulate gland openings in anterolateral corners; pygidium lacking apical stria; abdominal ventrite 1 with one or two lateral striae; ventrites 2–5 with or without posterior marginal striae. ***Legs*:** Protrochanter with seta; protibia lacking teeth, but with 8–10 stout marginal spines; protibial spurs short, strong; protarsus with fine ventral spines, pretarsal claws simple and equal; meso- and metatrochanters lacking setae; meso- and metatibiae nearly parallel-sided, the former with few weak marginal spines; meso- and metatarsomeres with single pair of apicoventral setae. ***Male*:** Eighth tergite with accessory sclerites, shallowly narrowly incised at subtruncate apex, with basal membrane attachment distad basal emargination; ventral apodemes of 8^th^ tergite broadly rounded, not meeting at midline; 8^th^ sternite approximately parallel-sided, halves not joined along midline, with apical guides gradually more strongly elevated toward apices; 9th tergite with median emargination deep, ventral apodemes situated just behind midpoint, strongly toothed; spiculum gastrale (9^th^ sternite) narrowed in distal two-thirds, with thin apical arms and short median apical flanges; halves of 10^th^ tergite well developed, separated along midline. ***Female*:** Eighth tergite forming a single, apically emarginate plate; 8^th^ sternite divided into two lateral plates, with thin, separate basal baculi which are articulated with the disk of S8; 9^th^ sternite present, elongate, connected to apex of S8; tenth tergite present, without basal apodemes; valvifers elongate, enlarged basally; coxites with two strong and one weak inner tooth; gonostyle present, free, setose; bursa copulatrix small; spermatheca short, sclerotized, forming a ventral concave disk over oviduct; spermathecal gland attached at base of spermatheca, elongate, gradually expanded to apex.

#### Distribution.

The distribution of the species of this genus is interestingly discontinuous, with three species concentrated in subtropical South America, and a single species from Central America, with few records from the northern half of South America.

#### Key to species

**Table:** 

1	Postmetacoxal striae of the first ventrite joined in an arch along the anterior margin (Fig. 4A); lateral metaventral stria terminating about one-third metaventral length behind the mesometaventral suture (Fig. 4A); frontal stria descending onto epistoma only, without stria across anterior margin of frons (Fig. 3A); southern Brazil, Paraguay, Argentina	*Kaszabister barrigai* sp. n.
–	Postmetacoxal striae not joined in an anterior arc; lateral metaventral stria reaching (Fig. 4B, D) or nearly reaching (Fig. 4C) mesometaventral suture; frontal stria variable	2
2	Abdominal ventrites 2–5 lacking posterior marginal stria (Fig. 4D); southern Mexico to Costa Rica	*Kaszabister carinatus* (Lewis, 1888)
–	Abdominal ventrites 2–5 with posterior marginal stria (Figs 4A-C)	3
3	Frontal stria complete and evenly arcuate across frons (Fig. 3B); epistoma convex; lateral stria of metaventrite reaching mesometaventral suture (Fig. 4B); Peru, Brazil, Uruguay, Argentina	*Kaszabister ferrugineus* (Kirsch, 1873)
–	Frontal stria not evenly arcuate, descending onto epistoma as a weak marginal carina (Fig. 3C); epistoma depressed; lateral stria of metaventrite abbreviated about one-fourth behind mesometaventral suture (Fig. 4C); southern Brazil, Uruguay, Argentina	*Kaszabister rubellus* (Erichson, 1834)

### Kaszabister barrigai sp. n.

urn:lsid:zoobank.org:act:03F0FB0E-8486-4E3A-A744-A242165F2C9C

http://species-id.net/wiki/Kaszabister_barrigai

Figs 1 , 2A , 3A , 4A , 6A–B , 7 (map)

#### Type material.

**Holotype:** male: **“**BRAZIL: State of Mato Grosso, Rondonópolis Co., Rondonópolis” / “R. Beasley, 14.VII.72, Floated from ant nest #169” / “Caterino/Tishechkin Exosternini Voucher EXO-00255 / HOLOTYPE *Kaszabister barrigai*Dégallier et al. des. 2012”; deposited in FSCA. **Paratypes** (109): **ARGENTINA: Cordoba**: Alta Gracia, La Granja, Sierras de Córdoba, 10.i.1925 (1: MACN); same locality and date, “debajo de uma piedra en el medio de las *Solenopsis*” (1: MACN); **ARGENTINA:** “on grapes (fruit) from Argentina, Kennedy, N.Y., 22.iv.1935” (1: NMNH); **BRAZIL: Mato Grosso:** Mato Grosso, Buriti, 20.vii.1972, floated from ant nest (3: FSCA); Mato Grosso, Cáceres, 5.vii.1972, floated from ant nest (6: FSCA); Mato Grosso, Cuiabá, Parque de Exposição, 30.v.1972, floated from ant nest (7: FSCA); same label data but 1.vi.1972 (12: FSCA); same label data but 6.vi.1972 (5: FSCA); same label data but 7.vi.1972 (3: FSCA); same label data but 13.vi.1972 (1: FSCA); same label data but 14.vi.1972 (4: FSCA); same label data but 14.vi.1972 (6: FSCA); same label data but 20.vi.1972 (13: FSCA); same label data but 22.vi.1972 (1: FSCA); Mato Grosso, Fazenda of Augusto Miller, 10 km N Chapada dos Guimarães, 21.v.1972, floated from ant nest (1: FSCA); Mato Grosso, Mato Grosso Co., 25.vii.1972, floated from ant nest (6: FSCA); Mato Grosso, Poconé, 23.iv.1972, floated from ant nest (9: FSCA); Mato Grosso, Pontes e Lacerda, 3.vii.1972, floated from ant nest (1: FSCA); 12 specimens from Mato Grosso, Rondonópolis, 14.vii.1972, floated from ant nest (12: FSCA); same label data but 15.vii.1972 (2: FSCA); Mato Grosso, Rosario Oeste, 13.vii.1972, floated from ant nest (3: FSCA). **São Paulo:** São Paulo, São Paulo, xii.1972, from fire ant nests (6: FSCA) ; São Paulo, Vargem Grande [do Sul], 1.xii.1972 (1: FMNH, 2: NMNH); São Paulo, Vargem Grande do Sul, 17.iii.1972, nest of *Solenopsis saevissima* gp. (1: FMNH); São Paulo, Varzea Grande, 9.ii.1972 (1: FSCA); same label data but 1.xii.1972 (2: FSCA). **PARAGUAY: San Pedro:** San Pedro, Cororó, x.1979 (1: CHND).

#### Diagnostic description.

Length 1.9–2.3mm; width 1.5–1.7mm;Frontal stria descending onto epistoma as a strong carina, epistoma strongly depressed, depression broader than in *Kaszabister rubellus*; fourth dorsal elytral stria present in apical half to two-thirds; inner subhumeral elytral stria present in apical two-thirds; fifth elytral stria generally absent; sutural stria present in apical half; elytral ground punctures denser and more uniformly distributed; mesometaventral stria arched forward to between one-half to one-third from anterior mesoventral margin; lateral metaventral stria strongly abbreviated mediad, ending about one-third from mesometaventral margin; inner postmetacoxal striae forming a complete, narrow arc across anterior margin of abdominal ventrite 1; abdominal ventrites 2–4 with apical marginal stria. ***Male*:** Aedeagus narrow, elongate, swollen toward base, flattened apically in lateral view.

#### Distribution.

Known principally from the Brazilian states of Mato Grosso and São Paulo, with one record each from Argentina and Paraguay, and a single specimen found in an Argentinian grape shipment at Kennedy Airport, New York, USA.

#### Biology.

While the majority of the type specimens say merely that they were collected from ‘ant nest’, there is little doubt that the hosts were *Solenopsis* Westwood, probably *Solenopsis invicta* Buren, 1972, the focus of the work of collectors Lennartz and Whitcomb (Allen et al. 1974, Buren et al. 1974). Other records specify ‘fire ants’, *Solenopsis* sp., and ‘*Solenopsis saevissima* group’ as hosts.

#### Etymology. 

This species is named for the notable Chilean collector Juan Enrique Barriga-Tuñón, who provided us with one of the first known examples of the species.

#### Remarks.

Specimens labelled as ‘São Paulo, Varzea Grande’ most probably came from Vargem Grande do Sul in the same state. We were not able to find any place called Varzea Grande in São Paulo. On the other hand, there is a town of this name that is a part of greater Cuiabá, the capital of Mato Grosso, where the fire ant researchers who collected specimens from Mato Grosso and São Paulo were apparently based. But given that we have seen specimens collected on the same day, 1.xii.1972, in ‘São Paulo, Vargem Grande’ (3) and ‘São Paulo, Varzea Grande’ (1), we suspect confusion and mislabeling for the latter locality.

### Kaszabister carinatus

(Lewis, 1888)

http://species-id.net/wiki/Kaszabister_carinatus

Figs 2B–E , 3D , 4D , 6G–H , 9 (map)

Phelister carinatus Lewis, 1888: 194.
Kaszabister carinatus : Mazur (1997: 31).

#### Type material.

Holotype (“one example”; Lewis, 1888) of undetermined sex: “Cerro Zunil, 4–5000 ft. Champion”/ “Sp. figured.” / “ B.C.A., Col.,II,(1). *Phelister*” / “*Phelister carinatus* Lewis Type” / “Type” [red circle] / “HOLOTYPE N. Dégallier 2007”; BMNH.

**Other material:** **MEXICO: Chiapas:** Mpio. Trinitaria, Lagunas de Montebello, 4–31.viii.1991, FIT (1: FMNH); **Michoacan**: San Jose Purua, vi.1965 (1: CHSM); ‘Mexico, A.G.’ (1: BMNH). **COSTA RICA: Alajuela:** RNVS Caño Negro, 18–30.xi.1992, 5–28.ii.1995 (2: INBI); **Guanacaste:** Est. Los Almendros, 4–16.ix.1994 (1, INBI); **Heredia:** Est. Biol. La Selva, 2.5 km S Puerto Viejo, 24.vi.2003, at light (1: EMEC); **Limón:** Cerro Cocori, iii.1993, iv.1993, v.1993 (3: INBI); Rio Sardinas, RNFS Barra del Colorado, 6–14.iv.1994 (1: INBI); Sect. Cerales de la Rita, 3 km N. del Puente Rio Suerte, Ruta Puerto Lindo, iii.1996 (1: INBI); Reventazon Evene, Hamburg Farm, 2.i.1932 (1: NMNH); P.N. Tortuguero, Est. Cuatro Esquinas, ix.1992 (1: INBI); **Puntarenas:** P.N. Corcovado, Est. Sirena, Send. Espavel, 19.iv.2001 (1: INBI); **San Jose:** San Jose, ix.1935 (1: CHND).

#### Diagnosis.

Frontal stria continuous across front and connected to epistomal striae, epistoma flat; fourth dorsal elytral stria complete and arched to suture; inner subhumeral elytral stria usually present only in apical half; fifth and sutural elytral striae weak, often just series of punctures, but generally present in apical one-third; elytral ground punctures sparser and markedly reduced laterad fourth elytral stria; mesometaventral stria arched forward to about one-half from mesoventral margin; lateral metaventral stria reaching mesometaventral margin, but distinctly mediad junction of mesometaventral and postmesocoxal striae; inner postmetacoxal striae somewhat variable, but never forming a complete arc across anterior margin of abdominal ventrite 1; abdominal ventrites 2–4 completely lacking apical marginal stria; aedeagus of moderate width, more strongly tapered basally than apically, with ventral curvature only marked nearer apex.

#### Distribution.

This species is restricted to Central America, from southern Mexico to Costa Rica.

#### Remarks.

No specimens bear any host data.

### Kaszabister ferrugineus

(Kirsch, 1873)

http://species-id.net/wiki/Kaszabister_ferrugineus

**Figure 5.**
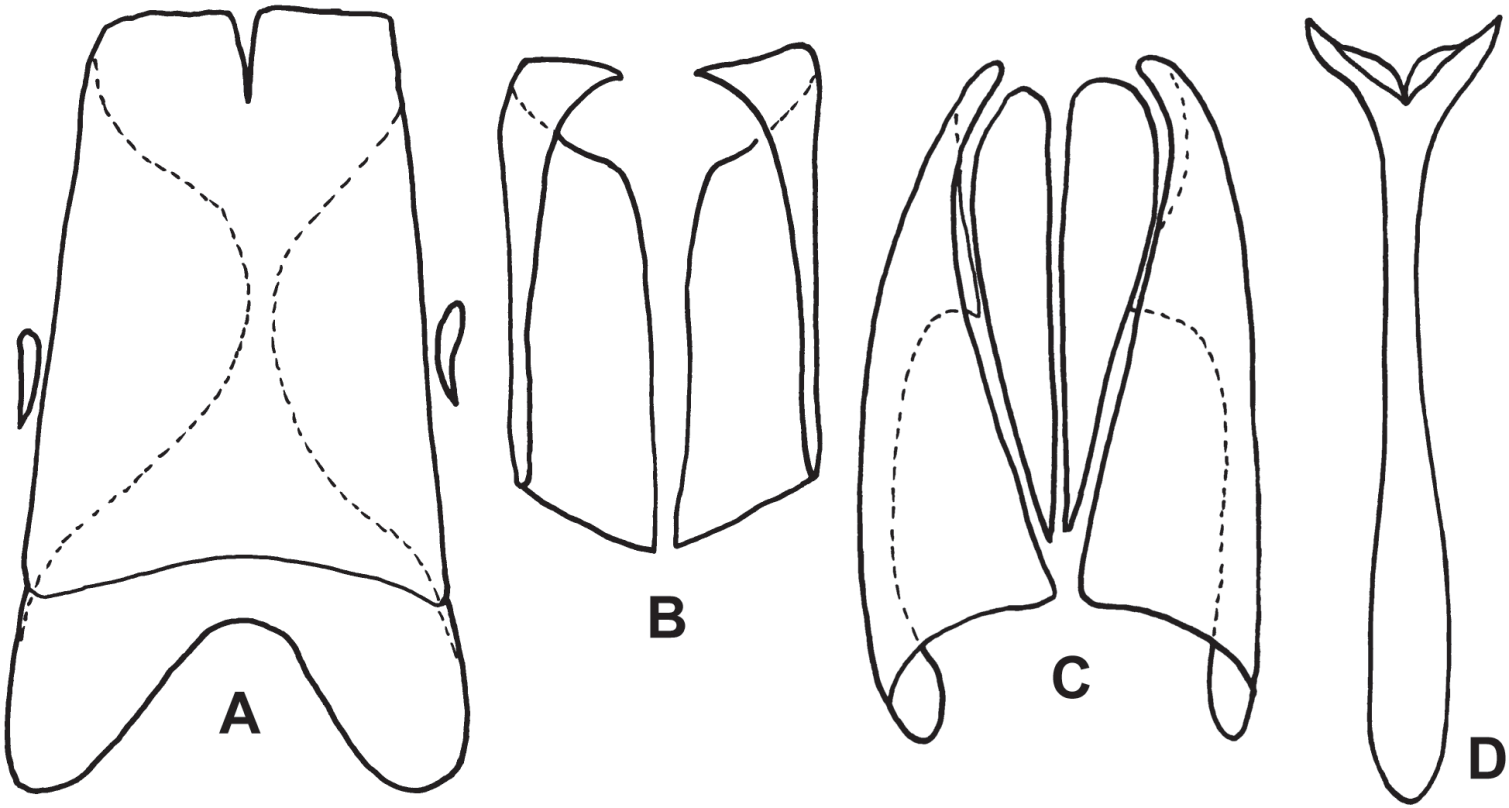
Male genitalia of *Kaszabister ferrugineus*. **A** 8^th^ tergite (with accessory sclerites) **B** 8^th^ sternite **C** 9^th^ and 10^th^ tergites **D** Spiculum gastrale (=9^th^ sternite).

**Figure 6.**
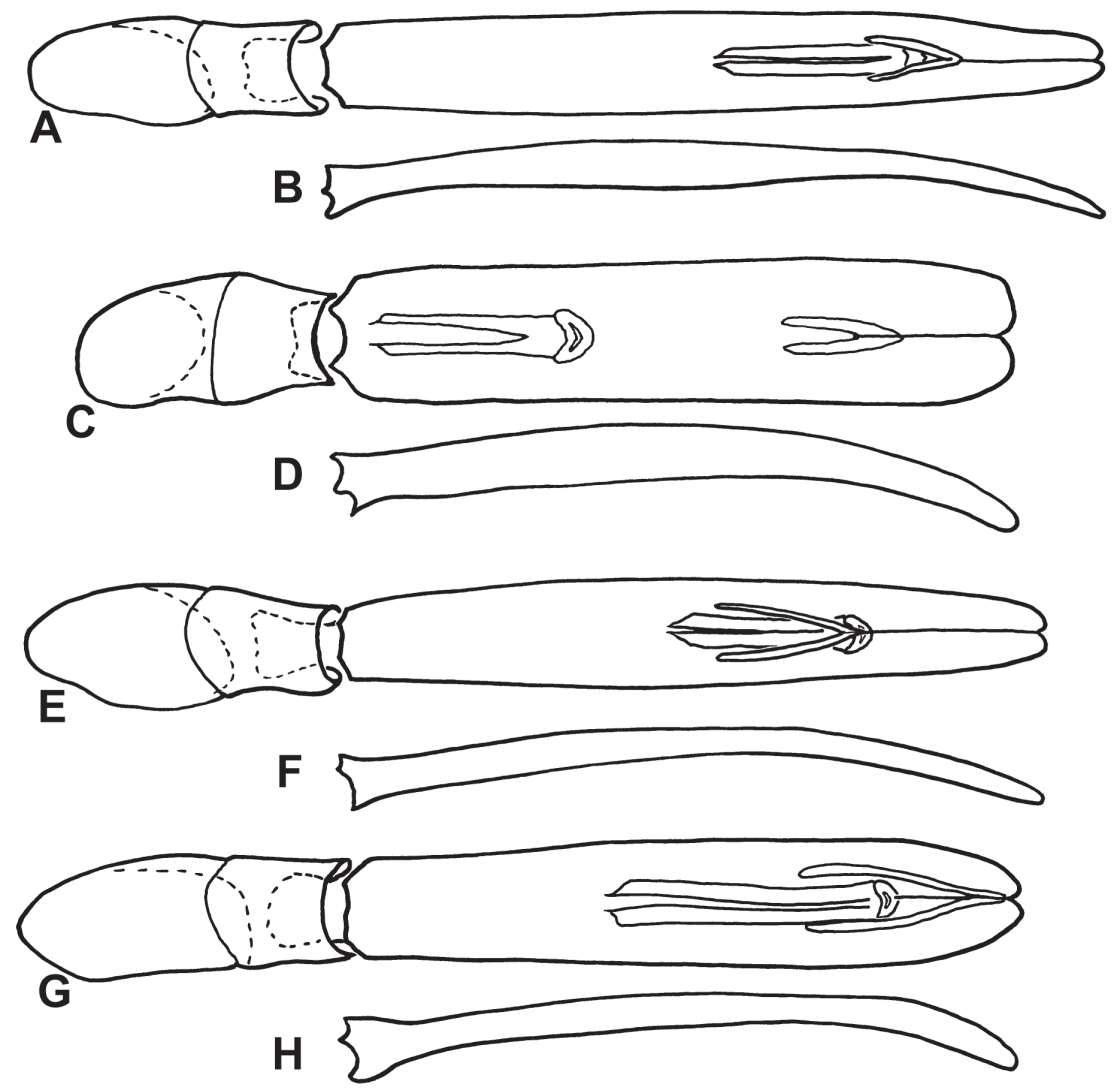
Male genitalia of *Kaszabister*. **A** Dorsal view of aedeagus of *Kaszabister barrigai* **B** Lateral view of aedeagus of *Kaszabister barrigai* **C** Dorsal view of aedeagus of *Kaszabister ferrugineus* **D** Lateral view of aedeagus of *Kaszabister ferrugineus* **E** Dorsal view of aedeagus of *Kaszabister rubellus* **F** Lateral view of aedeagus of *Kaszabister rubellus* **G** Dorsal view of aedeagus of *Kaszabister carinatus* **H** Lateral view of aedeagus of *Kaszabister carinatus*.

Figs 3B , 4B , 5 , 6C–D , 8 (map)

Epierus ferrugineus Kirsch, 1873: 137.
Phelister ferrugineus : Lewis (1905: 47).
Kaszabister ferrugineus : Mazur (1997: 31).
Phelister marginicollis Lewis, in litteris: Bruch (1914: 309).
Phelister marginicollis Bruch, 1914 (sic!): Mazur (1997: 31), as *nomen nudum*.

#### Type material.

Lectotype, herein designated for the purposes of establishing a unique and unambiguous type, as the original description omitted any indication of number of specimens studied: of undetermined sex: [Peru:] “Pozuzu, M. Kirsch” / “*Epierus ferrugineus*” / “Staatl. Museum für Tierkunde, Dresden” / “LECTOTYPE Dégallier & Mazur, 2007” / “*Kaszabister ferrugineus* (Kirsch, 1873); SMTD.

#### Other material.

**ARGENTINA: Buenos Aires:** Buenos Aires, 30.vii.1917, 9.vii.1923, 5.viii.1923 (4: MACN, NMNH); La Plata, viii.1912, nest of *Solenopsis saevissima* (2: NMNH); Rosas – F. C. Sud (7: NMNH); San Fernando, viii.1960 (1: NMNH). **Mendoza:** Mendoza, 20.x.1907 (1: ZMHB). **BRAZIL:** ‘**Bahia**’ (4: ZMHB). **Distrito Federal:** Brasilia, IBGE Ecological Reserve, 24.vi.1987, 13.v.1987, 7.vi.1987, 20.i.1998 (6: CIZUB, CHAT, CHND). **Mato Grosso:** Arenapolis, 22.viii.1972 (23: FSCA); Cáceres, 5.vii.1972 (1: FSCA); Cuiabá, 19.vi.1972 (1: FSCA); Mato Grosso Co., 25.vii.1972 (1: FSCA); Poconé, 23.iv.1972 (1: FSCA); Rondonópolis, 14–15.vii.1972 (3: FSCA); Rosario Oeste, 14.vii.1972 (1: FSCA). **Pará:** Belém, 2.vi.1985, flotation of the *Solenopsis saevissima* nest (identified by W. L. Overal) (1: CHND). **Rondônia:** Guajará Mirim, 1.ix.1972 (1: FSCA). **São Paulo:** São Simão, Usina Sta. Clara, 5.ix.1973 (3: FMNH). **URUGUAY: Artigas:** Ruta 30, km 45, 12.xii.1962, nest of *Solenopsis saevissima* (1: CHSM).

#### Diagnosis.

Frontal stria straight to evenly arcuate across front, not descending onto epistoma; fourth dorsal elytral stria complete and arched to suture; inner subhumeral elytral stria present in apical two-thirds; fifth elytral stria variable, from absent to present in apical one-half; sutural stria present in apical half; elytral ground punctures sparser and markedly reduced laterad fourth elytral stria; mesometaventral stria arched forward to about one-third from mesoventral margin; lateral metaventral stria reaching mesometaventral margin, nearly or fully meeting mesometaventral stria and postmesocoxal stria; inner postmetacoxal striae not forming a complete arc across anterior margin of abdominal ventrite 1, ending close to metacoxa; abdominal ventrites 2–4 with apical marginal stria; aedeagus rather short, parallel-sided, with apex subtruncate.

#### Distribution.

Known from Argentina (Mendoza, Buenos Aires), Brazil (Bahia, Distrito Federal, Mato Grosso, Pará, Rondônia and São Paulo), Peru (Huanuco) and Uruguay (Artigas).

**Figure 7.**
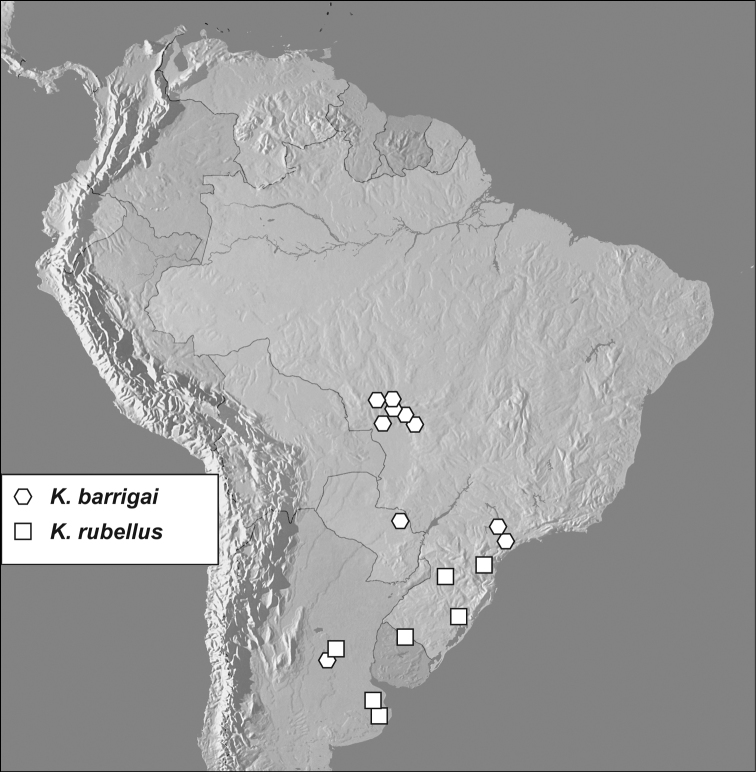
Map showing distributional records of *Kaszabister barrigai* and *Kaszabister rubellus*.

**Figure 8.**
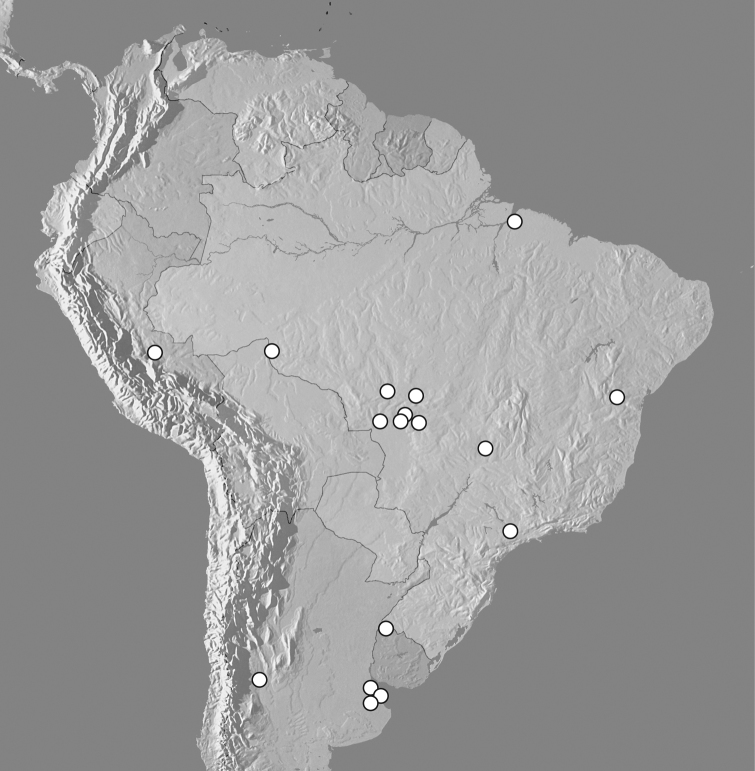
Map showing distributional records of *Kaszabister ferrugineus*.

**Figure 9.**
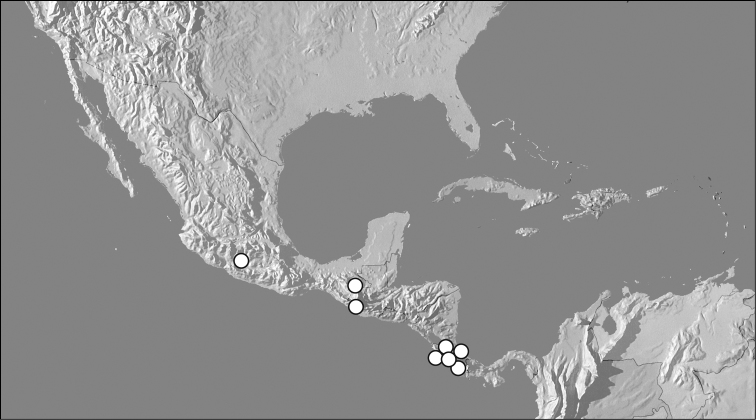
Map showing distributional records of *Kaszabister carinatus*.

#### Biology.

As for *Kaszabister barrigai*, many records of *Kaszabister ferrugineus* from ‘ant nests’ almost certainly refer to *Solenopsis invicta* as host, while a few other labels specify *Solenopsis saevissima* (Smith). One specimen from Bahia in the Lewis collection bears a label “with an *Aphaenogaster*” (Formicidae). However, no host specimen is present for verification.

#### Remarks.

Mazur (1997) cites “*Phelister marginicollis* Bruch, 1914” as “nom. nud. - syn. nov., Wenzel in litt.”. Bruch (1914: 309) cites this as “*Phelister marginicollis* Lew. in litteris”. This appears to have been a species that Lewis intended to describe based on Bruch’s specimens, and he indicated such to Bruch. Specimens labeled “*Phelister marginicollis* Lewis” are present in the BMNH and NMNH. However, for whatever reason, the species was never formally published, so it is indeed a nomen nudum. In Bruch’s later works (e.g., 1935) he apparently realized that the species was not properly described and omitted it from his catalog.

### Kaszabister rubellus

(Erichson, 1834)

http://species-id.net/wiki/Kaszabister_rubellus

Figs 3C , 4C , 6E–F , 7 (map)

Epierus rubellus Erichson, 1834: 163.
Kaszabister rubellus : Mazur (1984: 304).
Kaszabister mahunkai Mazur, 1972: 189. Synonymized by Mazur (1984).

#### Type material.

***Epierus rubellus*: Lectotype** herein designated for the purposes of establishing a unique and unambiguous type, as the original description omitted any indication of number of specimens studied: of undetermined sex: “*rubellus* Er., Carap[ava]. Sellow” / “48997” / “Zool. Mus. Berlin” / “*Kaszabister rubellus* (Erichson) LECTOTYPE N. DEGALLIER”; ZMHB. ***Kaszabister mahunkai*: holotype:** “Hungarian Soil-Zool. Exp., ARGENTINA: Prov. Córdoba, Fanti, Sierra de Córdoba, 11.I.1966” / “Nr. P-B.325 leg.Mahunka” / “Holotype 1972. *Kaszabister mahunkai* Mazur.” / “HOLOTYPUS” / “Compared with META-Types *Epierus rubellus* Er., R. L. Wenzel ‘73” / “*Kaszabister rubellus* (Er.) (= *mahunkai* Mazur), RLW ‘73” (HNHM).

#### Other material.

**ARGENTINA: Buenos Aires:** Balcarce, iv.1957 (1: NMNH); Rosas – F.C. Sud, with *Solenopsis* (6: NMNH). **BRAZIL: Paraná:** Rio Negro, with *Solenopsis* (1: BMNH); **Rio Grande do Sul:** Vallée de la Ferradura, Canela, 20.x.1989, from nest of *Solenopsis* sp. (1: CHND); **Santa Catarina:** Nova Teutonia, v.1937 (1: FMNH); 31.x.1948, with *Solenopsis* (2: FMNH); 2–3.xi.1948, with *Solenopsis* (2: FMNH); 11–14.xi.1948, with *Solenopsis* (2: FMNH); xii.1948 (1: FMNH); 6.vi.1950 (1: FMNH); 6.vii.1950 (1: FMNH); 2.viii.1950, with *Solenopsis* (4: FMNH); 26.viii.1950 (5: FMNH); 28.viii.1950, with *Solenopsis* (4: FMNH); 1.ix.1950 (1: FMNH); 3.ix.1950, with *Solenopsis* (1: FMNH); 6.ix.1950, with *Solenopsis* (2: FMNH); 7.ix.1950, with *Solenopsis* (1: FMNH); 18.ix.1950 (1: FMNH); 20.ix.1950, with *Solenopsis* (1: FMNH); 23.ix.1950 (1: FMNH); 4–5.x.1950, with *Solenopsis* (2: FMNH); 21.vii.1951 (1: FMNH); 24.vii.1951, with *Solenopsis* (1: FMNH); 31.vii.1951, with *Solenopsis* (8: FMNH); 17.viii.1951 (4: FMNH); 22.viii.1951 (1: FMNH); 5.ix.1951 (1: FMNH); 4.x.1951 (4: FMNH); 6–30.viii.1951, with *Solenopsis* (5: FMNH); 10.iv.1952, with *Solenopsis* (1: FMNH); 18.iv.1952, with *Solenopsis* (3: FMNH); 10.v.1952, with *Solenopsis* (1: FMNH); 10.v.1952, with *Solenopsis* (FMNH); 30.vii.1952, with *Solenopsis* (5: CHPK, CHSM, FMNH); 3.viii.1952, with *Solenopsis* (1: FMNH); 7.viii.1952, with *Solenopsis* (2: FMNH); 15.viii.1952 (1: FMNH); viii.1952, with *Solenopsis* (48: FMNH); ix.1952 (1: FMNH); vi.1954 (2: CHSM); vii.1954, (2: CHSM); xi.1956 (1: FMNH); ‘**Bresil**’ (1: MNHN). **URUGUAY: Rivera:** Rivera, Escuela Agraria, 18.ii.1962, nest of *Acromyrmex lundii* (Guérin-Méneville) (1: FMNH).

#### Diagnosis.

Frontal stria descending onto epistoma as a weak carina, epistoma moderately depressed, depression narrower than in *Kaszabister barrigai*; fourth dorsal elytral stria present in apical half to two-thirds; inner subhumeral elytral stria present in apical two-thirds; fifth and sutural elytral striae strongly reduced or absent; elytral ground punctures sparser and markedly reduced laterad fourth elytral stria; mesometaventral stria somewhat variable, arched forward to one-half to one-third from mesoventral margin; lateral metaventral stria weakly abbreviated mediad, ending about one-fourth to one-fifth metaventral length from mesometaventral margin, mesometaventral stria continuous with postmesocoxal stria; inner postmetacoxal striae nearly forming a complete arc across anterior margin of abdominal ventrite 1 (though often evanescent at very middle), this arc broader (closer to coxae) than in *Kaszabister barrigai*; abdominal ventrites 2–4 with apical marginal stria; aedeagus narrow, approximately evenly tapered basally and apically; flatter (in lateral view) than in other species.

#### Distribution.

Known from Brazil (Paraná, Rio Grande do Sul, Santa Catarina), Argentina (Buenos Aires, Córdoba), Uruguay (Rivera).

#### Biology.

Most specimens from Santa Catarina bear labels indicating collection with unspecified *Solenopsis*. The singleton from Uruguay indicates collection from a nest of *Acromyrmex lundii*.

#### Remarks.

The synonymy of *Kaszabister mahunkai* Mazur with *Epierus rubellus* Erichson was originally designated by Mazur (1984) (citing “Wenzel, in litt.”). We have not studied the type of *Kaszabister mahunkai* first-hand, but Ottó Merkl of the HNHM very kindly compared the type specimen with our descriptions, keys and figures, and had no doubt that the synonymy is valid. His study also confirmed that Wenzel had compared types of *Kaszabister mahunkai* and *Epierus rubellus* side-by-side in coming to his original conclusion that the two were conspecific.

## Discussion

Collecting records indicate that most of the species are strongly or exclusively associated with fire ants in the *Solenopsis saevissima* species group (excepting *Kaszabister carinatus* for which no ecological data is available). Most specimens appear to have been washed out of host mounds, and there have been no reported observations of any behavioral interactions of the *Kaszabister* beetles and their hosts. As all known histerids are predaceous (Kovarik and Caterino 2005), there can be little doubt that the beetles prey on their hosts, probably the larvae and pupae. From some focused collecting it appears that the density of these beetles can be relatively high, and it’s conceivable that they provide a significant level of natural control of fire ant populations. Significant interest and resources have focused on introduction of parasitic flies as natural enemies of invasive fire ants in the United States (Callcott et al. 2011). None, to our knowledge, has considered predaceous myrmecophilous beetles. It should also be noted here that, although not as common a host as *Eciton* Latreille, *Solenopsis* does host other neotropical histerid genera, including *Hippeutister* Reichensperger and *Procolonides* Reichensperger (Helava et al. 1985, Kovarik and Caterino 2005, Caterino and Tishechkin 2008).

Myrmecophily is a common phenomenon in Histeridae, with two entire large subfamilies (Haeteriinae and Chlamydopsinae) composed almost entirely of myrmecophiles (Kovarik and Caterino 2005). Outside these major groups, however, there have been numerous independent acquisitions of myrmecophilous habits in nearly all other major taxa. In Exosternini myrmecophily has arisen in both Old and New World genera, including *Paratropus* Gerstaecker, *Coelocraera* Marseul, *Phelister* Marseul, *Pseudister* Bickhardt and *Tribalister* Horn. Specialized myrmecophily is often associated with a suite of morphological specializations (Helava et al. 1985, Kovarik and Caterino 2005). Of these, only exaggerated striae/carinae (frontal, pronotal, elytral) present themselves in *Kaszabister*.

*Kaszabister* exhibits a largely disjunct distribution, with very few records from a large area of northern South America. At this point it seems most likely that this relates to sampling effort. The vast majority of existing specimens have resulted from direct sampling of *Solenopsis* colonies, which has generally been focused on those areas with species that have become invasive elsewhere, primarily in southern Brazil. At the same time, fairly intensive passive trapping (flight intercept trapping and pitfall trapping) in some of these areas has resulted in no specimens (Vaz-de-Mello unpublished data, Flechtmann unpublished data). So although other parts of South America have seen significant trapping, by ourselves and others, there has been relatively little effort to collect in the appropriate ant colonies. So it remains to be seen whether *Kaszabister* is really more widespread but undetected in South America. This situation is very similar to that of *Hippeutister* (Histeridae: Haeteriinae), with species known from Central America and southern Brazil (Caterino and Tishechkin 2008). Dedicated collecting efforts in *Solenopsis* nests elsewhere may well turn up additional species in both these groups.

An extensive collecting of *Kaszabister* in host ant colonies in 1972 in Mato Grosso has revealed some interesting facts about sympatry and syntopy of its species. Out of nine localities where *Kaszabister barrigai* was collected, at six *Kaszabister ferrugineus* was also found. Moreover, collecting colony codes reveal that both species have been collected in the same nests at Cáceres, Cuiabá, Mato Grosso Co., and Poconé. At the six localitites where both species were found *Kaszabister barrigai* was much more common, outnumbering *Kaszabister ferrugineus* by 100 to eight specimens. The situation at another locality, Arenapolis, was completely reversed: 23 specimens of *Kaszabister ferrugineus* were found in three nests there but no *Kaszabister barrigai* specimens are known from that site. Although nothing else known about this remarkable syntopy, the abundance patterns are suggestive of either interspecific competition, or very fine habitat and/or host preferences. *Kaszabister ferrugineus* and *Kaszabister rubellus* are also known to co-occur in at least one location; both were collected at Rosas, Argentina, represented by seven and six specimens, respectively. However, due to poor labeling, it is unclear whether they coexist microsympatrically or simultaneously, e.g. in the same colony or at the same time. This would be interesting to explore further.

The phylogenetic position of *Kaszabister* within Exosternini has never been addressed. Ongoing analyses suggest a relationship with another inquilinous and enigmatic genus, *Mecistostethus* Marseul, together as sister or near sister to the large genus *Operclipygus* Marseul. However, some of the morphological characters these share may be convergences related to myrmecophily. Final analyses remain ongoing, and a confident result with respect to *Kaszabister* will probably have to await the availability of molecular sequence data.

## Supplementary Material

XML Treatment for Kaszabister

XML Treatment for Kaszabister barrigai

XML Treatment for Kaszabister carinatus

XML Treatment for Kaszabister ferrugineus

XML Treatment for Kaszabister rubellus
